# Overnight auto-adjusting continuous airway pressure + standard care compared with standard care alone in the prevention of morbidity in sickle cell disease phase II (POMS2b): study protocol for a randomised controlled trial

**DOI:** 10.1186/s13063-017-2419-0

**Published:** 2018-01-22

**Authors:** Jo Howard, April E. Slee, Simon Skene, Baba Inusa, Jamie Kawadler, Michelle Downes, Johanna Gavlak, Melanie Koelbel, Hanne Stotesbury, Maria Chorozoglou, Susan Tebbs, Subarna Chakravorty, Moji Awogbade, David C. Rees, Atul Gupta, Patrick B. Murphy, Nicholas Hart, Sati Sahota, Carol Nwosu, Maureen Gwam, Dawn Saunders, Vivek Muthurangu, Nathaniel Barber, Emmanuel Ako, Swee Lay Thein, Melanie Marshall, Isabel C Reading, Man Ying Edith Cheng, Fenella J. Kirkham, Christina Liossi

**Affiliations:** 1 Department of Haematology, Guy’s and St Thomas’ Hospitals NHS Foundation Trust, London, UK; 2 Comprehensive Clinical Trials Unit at UCL, London, UK; 3 Evelina Children’s Hospital, Guy’s and St Thomas’ Hospitals NHS Foundation Trust, London, UK; 4 London, UCL GOSH Institute of Child Health, 30 Guilford Street, London, WC1N 1EH UK; 5 Department of Child Health, University Hospital Southampton, Tremona Road, Southampton, SO16 6YD UK; 6 King’s College Hospital, London, UK; 7 King’s College London, London, UK; 8 Lane Fox Respiratory Unit, Guy’s and St Thomas’ Hospitals NHS Foundation Trust, London, UK; 9 University of Southampton, Southampton, UK; 10 Sickle Cell and Young Stroke Survivors Charity, London, UK; 11 Centre for Translational Cardiovascular Imaging, UCL Great Ormond Street Institute of Child Health, London, UK; 12 National Institutes of Health, Bethesda, MD USA; 13 University of New South Wales, Sydney, NSW Australia; 14 Research Design Service, University Hospital Southampton, Southampton, UK

**Keywords:** Sickle cell anaemia, Haemoglobin oxygen saturation, Auto-adjusting continuous positive airways pressure, Cancellation, Attention, Processing speed

## Abstract

**Background:**

In addition to pain, sickle cell anaemia (HbSS) complications include neurocognitive difficulties in attention and processing speed associated with low daytime and night-time oxygen saturation compounded by obstructive sleep apnoea (OSA). In the general population OSA is treated with continuous positive airways pressure (CPAP). The aim of this single-blind, randomised, controlled phase II trial is to compare auto-adjusting CPAP (APAP) with standard care to standard care alone in individuals with HbSS to determine whether the intervention improves attention and processing speed, brain structure, pain and quality of life.

**Methods/Design:**

Eligibility criteria include: ability to provide informed consent; age > 8 years; diagnosis of HbSS; and mean overnight saturation of < 90% for < 30% of the night (i.e. not meeting current criteria for overnight oxygen therapy). Key exclusion criteria are: overnight respiratory support; respiratory or decompensated cardiac failure; chronic transfusion; or contraindications to APAP therapy or magnetic resonance imaging (MRI).

Sixty individuals with HbSS (30 children and 30 adults) will be randomised to standard care + APAP or standard care alone for six months. Minimisation factors are: age group (8–11, 12–15, 16–22 and > 23 years); silent infarction on MRI; minimum overnight oxygen saturation > 90% or < 90%; and hydroxyurea use.

For APAP individuals, the intervention is administered at home. Adherence and effectiveness are recorded using software documenting hours of use each night and overnight oximetry. Participant support in terms of appropriate facemask and facilitating adherence are provided by an unblinded sleep physiologist.

The primary outcome is change in the cancellation subtest from the Wechsler scales. Secondary outcomes include general cognitive functioning, quantitative brain MRI, blood and urine chemistry, quality of life and daily pain via a smartphone App (GoMedSolutions, Inc) and, where possible MRI heart, echocardiography, and 6-min walk. These outcomes will be assessed at baseline and after six months of treatment by assessors blind to treatment assignment.

**Discussion:**

Altering oxygen saturation in HbSS may lead to bone marrow suppression. This risk will be reduced by monitoring full blood counts at baseline, two weeks, three months and six months, providing treatment as appropriate and reporting as safety events.

**Trial registration:**

ISRCTN46012373. Registered on 10 July 2015.

Protocol Version: 6.0 Date: 24th December 2015

Sponsor: University Hospital Southampton. Sponsor’s protocol code: RHMCHIOT53

## Background

Sickle cell anaemia (SCA; HbSS) is an inherited red blood cell disorder associated with painful crises, acute chest crisis, cardiac dysfunction and a high risk of clinical stroke and ‘silent’ infarction. Even in the absence of stroke, children and adults may develop cognitive deficits [1] that have a significant impact on quality of life. Processing speed appears be a particular problem in those with clinical and ‘silent’ cerebral infarction (SCI) [2, 3], but adults and children with SCA without SCI still show reduced speed of processing on the Wechsler scales [4–6], affecting other aspects of cognition [7]. Specific deficits in attention, working memory and other aspects of executive function are frequently reported [4, 8–11].

Low daytime and night-time oxygen saturation (SpO_2_), potentially exacerbated by sleep-disordered breathing, including snoring and obstructive sleep apnoea (OSA), are more common in SCA than in the general population [12]. As there is a paucity of population-based normative data for sleep physiology in children and adults, it is difficult to apply cut-offs for intervention in SCA, particularly as the threshold might be lower as acute hypoxic exposure causes sickle haemoglobin to polymerise [13]. When polysomnography is available, > 40% of unselected children with SCA have an obstructive apnoea hypopnoea index (OAHI) > 1, [12] and the majority have OAHI above the normal range [14]. As this is an expensive investigation, there are few data in unselected populations of adults with or without SCA, but in a recent study, all SCA adults with sleepiness or symptoms of sleep-disordered breathing, e.g. snoring, had an apnoea hypopnoea index of > 1 [15]. Compared with data from the general paediatric population, [16, 17] mean and/or minimum oxygen saturation on overnight pulse oximetry are lower in the majority of patients with SCA [12, 15, 18] (Howard et al., submitted).

In addition to the evidence for an association of oxygen desaturation with endothelial dysfunction, [19, 20] cerebrovascular disease [21] and stroke [22, 23], cardiac dysfunction [24] and hospital days for pain, [25] there is evidence for links with cognitive difficulties, [26, 27] in line with previous experimental and clinical evidence. Animal data show that intermittent hypoxia during sleep is associated with impaired spatial learning and hippocampal vulnerability [28]. In addition to the evidence for a link with cerebrovascular disease, [29] minimum oxygen saturation is associated with cognitive difficulties in OSA [30] in the adult general population and in other conditions, such as multiple sclerosis [31]. Magnetic resonance imaging (MRI) abnormalities have also been reported in relation to oxygen desaturation in SCA [32]. In 25 individuals with SCA but no evidence of SCI, a whole-brain voxel-wise MRI analysis of white matter (tract-based spatial statistics [TBSS]), showed a significant correlation of reduced daytime SpO_2_ and high radial diffusivity (RD) in the anterior corpus callosum (*p* < 0.05) [32]. The anterior corpus callosum contains fibres connecting the left- and right-hemisphere prefrontal cortex, underpinning important executive functions. These results suggest that chronic oxygen desaturation could play a vital role in the viability of frontal lobe white matter and so diffusion tensor imaging metrics may serve as biomarkers for monitoring therapeutic effects. Hippocampal volumetric deficits have been shown in children and adolescents with SCA [33]. In adults with OSA, cognition is compromised and hippocampal alterations seen on MRI have been reported; importantly, the effects of hypoxic exposure on cognition and hippocampal volume have been reported to be reversible [34].

In patients with SCA, reduced exercise tolerance is reported in daily living and observable during a 6-min walk test (6MWT). In addition, structural abnormalities on echocardiography [35] and cardiac MRI [36] are well recognised. There are, however, few published data on how cardiac function is related to sleep-disordered breathing [24] or changes during exercise [37] or whether these findings can be reversed.

There is therefore a case for exploring whether interventions to improve sleep-disordered breathing, OSA and nocturnal desaturation improve clinically relevant endpoints in SCA. Adenotonsillectomy is an invasive procedure which may not cure OSA [38]. Continuous positive airways pressure (CPAP) is standard treatment for OSA in adults but the potential discomfort of breathing against a continuous pressure of 10 cm water is not easy to justify unless there is polysomnographic evidence of frequent airway obstruction. For auto-adjusting continuous positive airways pressure (APAP), the continuous pressure is set to a more comfortable pressure of around 4 cm water and the pressure only rises to a preset maximum of, for example, 10 cm water when obstruction is detected by the machine, making it more appropriate for patients with only a few episodes of obstruction per night; [39] additional modalities may be effective for causes of sleep-disordered breathing other than OSA [40]. APAP is likely to improve minimum oxygen saturation by preventing desaturation in even minor degrees of airway obstruction. Although occasional adverse events (AEs) related to APAP are reported, we saw none in our previous pilot studies; the main problem with positive airways pressure interventions is non-compliance if the patient does not feel that the nuisance is worth any potential benefit.

In a small randomised trial of standard care vs APAP in children with SCA, all 12 were compliant for six weeks and both daytime and mean overnight oxygen saturation increased, the latter significantly [18]. A psychologist blind to treatment arm undertook five subtests of the Wechsler scales chosen to examine processing speed, attention and working memory. The primary endpoint, processing speed index, improved in the standard care as well as the treatment arm, possibly in part due to a practice effect. However, cancellation, a Wechsler subtest of visual attention as well as processing speed, showed statistically significant improvement only in the treatment arm [18]. In addition, in a pilot study (Prevention of morbidity in sickle cell disease, POMS 2a) (Howard et al., submitted) for this phase II study (POMS 2b), we compared safety and tolerability in children and adults for two alternative interventions: APAP and Nocturnal Oxygen Therapy (NOT). In a crossover design, after a week’s baseline, each intervention was used for one week, with a week-long washout between phases. Compared to baseline, individuals experienced small increases in daytime oxygen saturation and decreases in haemoglobin and for the week on both APAP and NOT, but there was no difference between interventions (Howard et al., submitted). Ten of 16 (62.5% [95% confidence interval = 38.6–81.5]) who completed qualitative interviews reported a preference for APAP (Howard et al., submitted). Practical advantages of APAP include the cost, size and portability of the APAP machine, an optional humidifier attachment to treat dry throat, an adjustable mask, optional addition of oxygen if required and capacity for remote compliance monitoring.

In the current POMS 2b Phase II study, ethical and practical considerations preclude blinding individuals and investigators, but the risk of bias in outcome assessment can be minimised by utilising cognitive and imaging endpoints assessed by blinded personnel. A single-blind design is justified as preliminary evidence suggests that treating OSA and improving oxygen saturation, for example, using overnight oxygen [41] or CPAP, [18, 42] might improve cognition and brain structure. APAP has a favourable risk–benefit profile; the main safety concern in altering oxygen saturation in SCA is the possibility of bone marrow suppression resulting in reductions in haemoglobin, red cell count, reticulocytes and erythropoietin [43].

### Aim

The objective of this single-blind, randomised, controlled phase II trial is to compare APAP with standard care in individuals with HbSS to determine whether six months of using the intervention improves attention and processing speed, brain and heart structure, pain and quality of life

## Methods and design

This is a single-blind randomised controlled phase II trial consisting of two separate cohorts of people with HbSS (children and adults) recruited through two London National Health Service hospitals in the UK (Guy’s and St Thomas’ NHS Trust and King’s College Hospital NHS Trust, both academic centres as part of King’s Health Partners). Though some pooled analyses may be conducted, the cohorts are independent and will be analysed separately. Informed consent/assent will be obtained from all participants in the study. After randomisation, individuals assigned to APAP will be provided with a REMstar® Auto System, which is an APAP device designed for the treatment of OSA and is supplied by Respironics. When set in the Auto‐APAP mode, the system will monitor breathing during sleep and automatically adjust the pressure to meet therapeutic needs. Individuals in the APAP arm will use this intervention nightly for six months. Individuals randomised to standard care will have treatment according to routine National Health Service procedure for sickle cell disease for six months post randomisation.

The intervention involves wearing a mask for several hours most nights, so it is difficult to justify a sham treatment arm of six months of overnight respiratory support. The outcomes will be assessed by study personnel blind to treatment arm so that although the family and some professionals involved in data collection know the treatment allocation, the assessors of endpoints, including cognitive function, imaging, pain frequency and quality of life, will not know the treatment assignment. All study personnel not directly involved in treatment administration and compliance will be blinded to randomisation assignment until the blind is broken for each cohort. Blinded study personnel include data managers and the trial statistician, as well as assessors of endpoints.

APAP dose reductions are permitted if required, e.g. in the case of AEs. Discontinuation of participants from the trial will occur if there is withdrawal of a child’s assent to participate or participant/parental/guardian withdrawal of consent to participate or loss to follow-up; wherever possible the primary and secondary endpoint will be collected.

### Eligibility and informed consent

Coordinators, principal investigators and the chief investigator, all appropriately trained, will obtain consent. Individuals will be recruited from haematology/sickle/respiratory clinics and will be eligible if they give informed consent or assent for children, are aged > 8 years with HbSS diagnosed by standard techniques (HPLC, IEF and MS) and have overnight oximetry showing mean overnight saturation of < 90% for < 30% of the night (i.e. they do not meet current recommended levels of overnight oxygen desaturation for referral for overnight oxygen therapy). Individuals will be excluded if they already have overnight respiratory support, if they have existing respiratory or decompensated cardiac failure or if they have any contraindications to APAP therapy. If participants present with sinus or middle ear infections, they are to be excluded until the infection has been treated and resolved. They may then be evaluated for trial eligibility.

### Randomisation

Central randomisation by computer by the company ‘Sealed Envelope’ will be used with a minimisation algorithm [44] incorporating a random element, stratifying by baseline oximetry minimum overnight saturation (<90% vs ≥ 90%), whether or not a participant is currently prescribed hydroxyurea, presence or absence of infarction on MRI and age group: 8–11 vs 12–15 years in children, and 16–22 vs ≥ 23 years in adults. To ensure maximum balance is achieved across the stratification factors, minimisation will be carried out on these factors separately. Individuals will be randomised to APAP + standard care or standard care alone. The coordinator will be informed of the allocation by email and will arrange for the respiratory physiologist to set up APAP at home for those randomised to APAP.

### Sample size

This study consists of two separate and independent cohorts. The first cohort will consist of 30 children (aged 8–16 years) randomised 1:1 to either APAP or standard of care. The second cohort will consist of 30 adults (aged ≥ 16 years) randomised 1:1 to APAP + standard care or standard care alone. Each cohort represents an independent trial with 90% power to detect a difference in cancellation rate at a significance level of 0.05. Since the cohorts are independent and each has a type I error allocation of 5%, no adjustments for multiple comparisons are needed. Any analyses pooling the child and adult cohorts will be considered exploratory.

In the previous pilot study in children, the results of the cancellation task within each subject group were normally distributed with standard deviation 2, resulting in a mean score difference between APAP treatment and standard care at six weeks of 2.6. Based on this finding, 24 evaluable participants (in each cohort) will provide 90% power to detect a difference in the primary outcome of 2.6 while preserving a significance level of 0.05 (two-sided). This calculation assumes that there is a moderate correlation of 0.5 between the baseline and follow-up measures, and accounts for model adjustment for baseline score [45]. Allowing for 20% withdrawal/loss-to-follow-up, a sample size of 30 individuals randomised 1:1 into two groups will have > 90% power to detect a difference of 2.6 points in cancellation using an analysis of covariance (ANCOVA) model to adjust for the baseline cancellation measure and minimisation factors. The same design characteristics will be used for the adult cohort and the child cohort. Though the values used in the sample size calculation were derived from a cohort of children, no pilot data for adults are available, and the mechanisms by which the treatment effect may be different for adults and children are currently unknown.

### Outcomes

The primary outcome is change from baseline to six months post randomisation in cancellation, a measure of visual attention and processing speed. Secondary outcomes are changes in cognition, pain intensity, quality of life, daytime oximetry, brain MRI and cardiac investigations. Health economic measures will determine quality of life measures in quality-adjusted life years (QALY) terms and the main cost drivers in terms of therapy resources, equipment and travel, etc., and any change in use of other resources, e.g. requirement for transfusion, opioids, other pain relief and hospital admissions to provide a preliminary estimate of the cost of overnight respiratory support compared to standard care. AEs and evidence of bone marrow suppression are key safety outcomes.

### Data collection

**Fig. 1:**
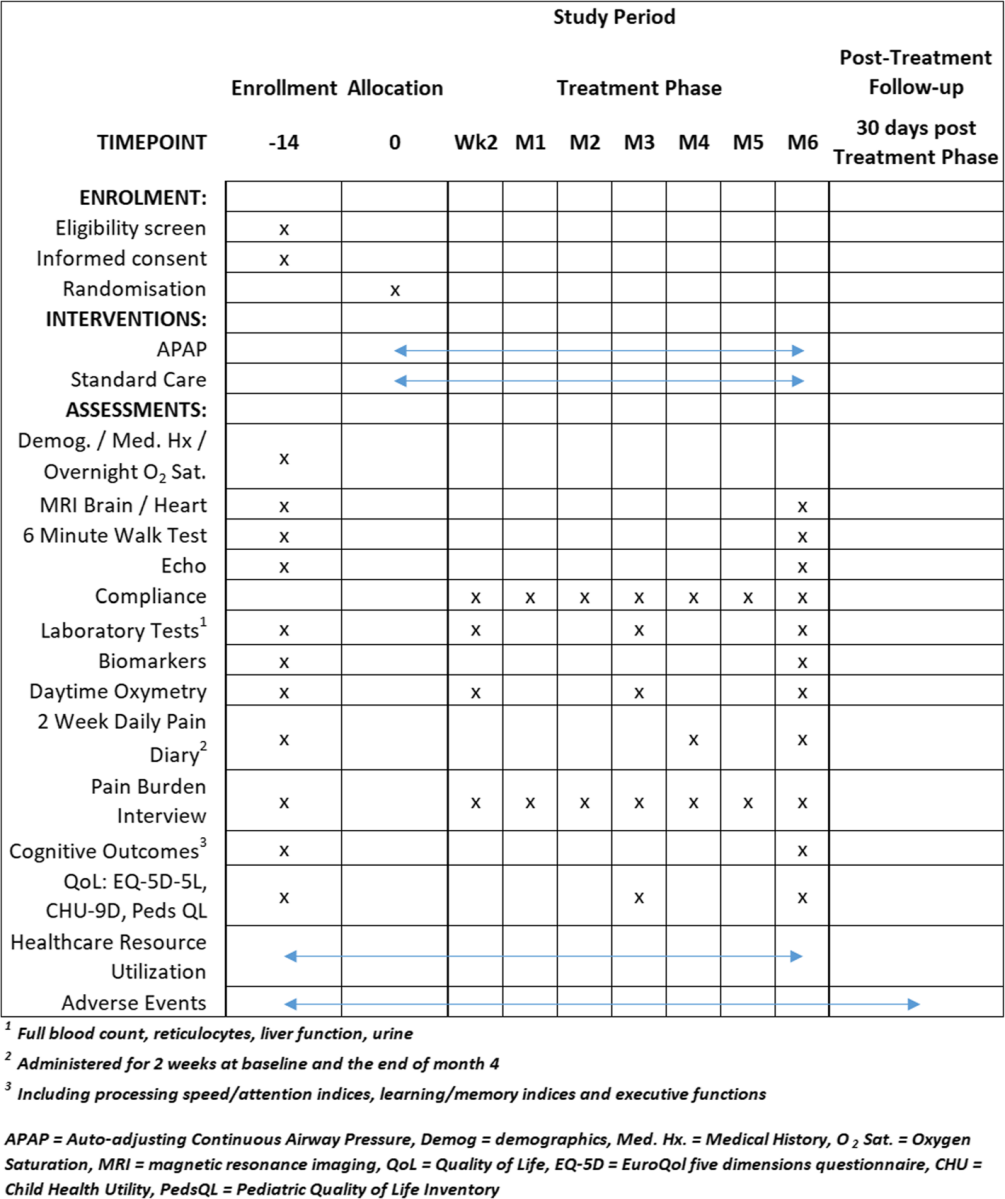
Data collection

Data collection is summarised in Fig. 1. Baseline assessments include demographics, medical history, concomitant medications, physical examination, sleep habits, [46] Epworth Sleepiness instrument [47] and measurements of pain burden [48] and daily pain as well as quality of life using the CHU-9D21 in children [49] and the Euroqol EQ-5D22 in adults [50] as well as the sickle module of the PEDS-QL [51]. Physiologic tests at baseline include a brain MRI and overnight oximetry, which are required for minimisation, as well as daytime oximetry and laboratory measures. The baseline cognitive outcomes assessment is also required before randomisation. Whenever possible, investigators are encouraged to perform heart MRI, echocardiography and a 6MWT.

**Fig. 2:**
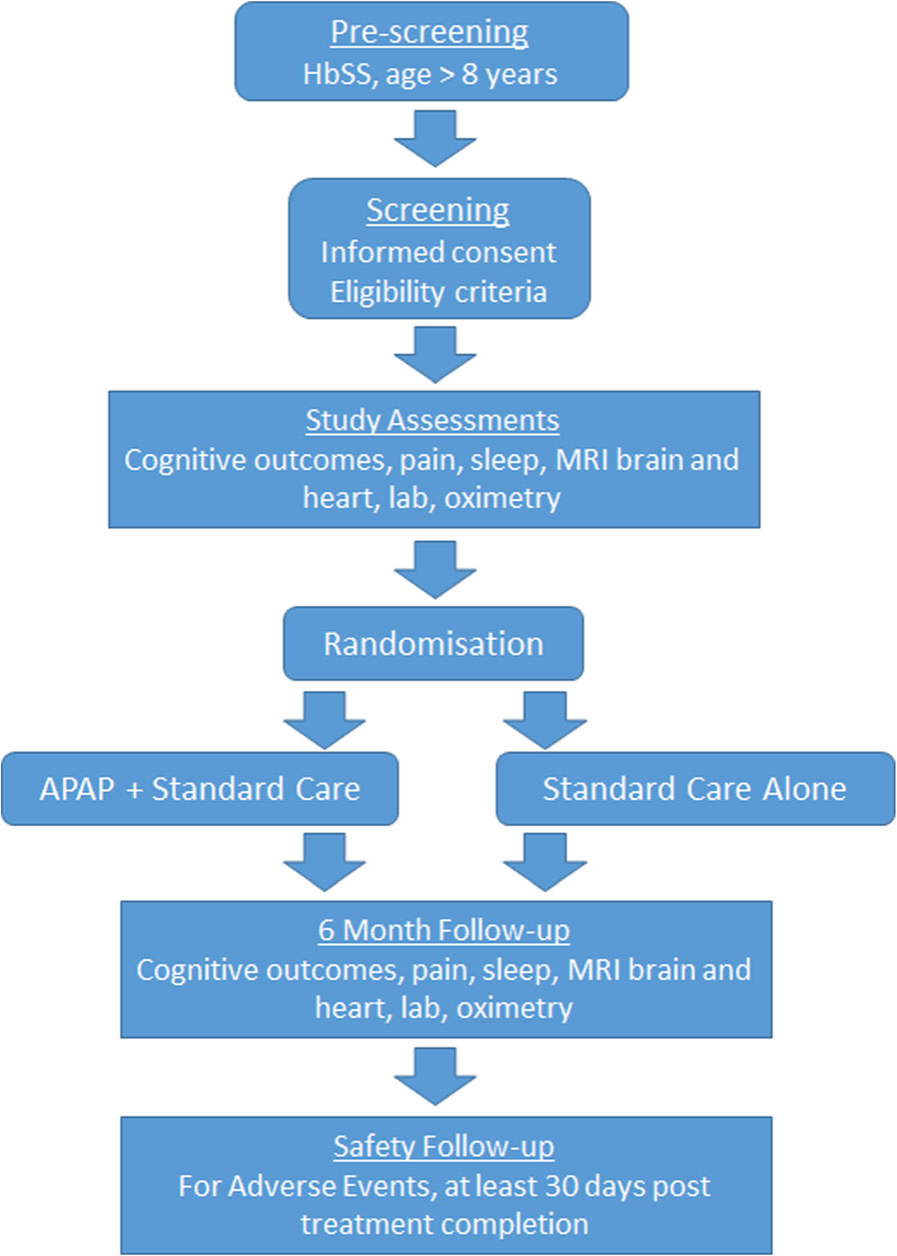
Study flow

Study flow from randomisation through follow-up is shown in Fig. 2. Two weeks after commencement of randomised treatment, individuals will have a face-to-face review for AEs and adherence, and will have daytime oximetry and blood and urine tests. Participants will also have a repeat overnight oximetry at home. Face-to-face assessments for AEs, adherence, daytime oximetry, blood and urine will also take place at months 3 and 6. At months 1, 2, 4 and 5, individuals will be contacted by the study coordinator for review of AEs, treatment adherence and administration of the sickle cell pain burden questionnaire [48].

At the end of the trial (month 6), participants will undergo end of study tests, including blood and urine tests, medical review, review of adherence and AEs, quality of life, executive function testing and MRI of the brain unless contraindicated. Whenever possible, echocardiography, a 6MWT and heart MRI will also be performed.

Data will be collected on paper case report forms to be stored in a locked cabinet and later double-entered into data management software password-protected and stored securely.

### Cognitive outcomes

Several studies have shown an association of nocturnal hypoxia and sleep-disordered breathing with cognitive impairment, including deficits in processing speed, visual attention, executive function and memory. Moreover, improvement in processing speed has been demonstrated in the general population with OSA following adenotonsillectomy [52] as well as in patients with SCA treated with APAP [18]. In light of these findings, a battery of cognitive tests will be used to track cognitive outcomes in the current study.

In the two weeks between recruitment and randomisation, and again at the end of the study, a psychologist blind to treatment arm will administer standardised tests applicable and comparable in children and adults, with demonstrated validity and reliability, to examine cognitive domains which may improve if overnight desaturation is relieved and sleep improves.

The baseline cognitive assessment will measure general cognitive ability using the Wechsler Abbreviated Scale of Intelligence (WASI), and processing speed, working memory and cancellation using the Wechsler Intelligence Scale for Children-IV ([WISC-IV], age < 16 years) or Wechsler Adult Intelligence Scale-IV ([WAIS-IV], age > 16 years). The Wechsler scales were chosen as they have normative scores validated in a representative sample, including ethnic minorities, in the UK, show strong reliability and validity, are widely used in the literature and are frequently used measure of cognitive function in sleep apnoea as well as SCA, with demonstrated sensitivity to dysfunction in SCA as well as to change following APAP [18]. Moreover, the Wechsler scales have an extensive empirical database, rigorous standardisation procedures and robust psychometric properties, including well-replicated validity, stability and reliability. The cancellation subtest can be administered across a wide age range with age-adjusted scaled scores that are in the range of 1–19. A scaled score of 8–12 is considered to be within the average range.

In addition, on the basis of previous research suggesting sensitivity to dysfunction in SCA, executive function will be assessed using the Tower and Sorting subtests of the Delis–Kaplan tests of Executive Function [D-KEFS], and via parent- (<16 years) or self- (>16 years) report using the BRIEF questionnaire. The BRIEF-questionnaire is sensitive to atypical variations in executive function (EF) development [53] and is the most commonly used screener questionnaire for executive dysfunction. It is either used as self- report for adults (i.e. 18 and 90 years) or given to the child’s parents (i.e. caregiver) to answer questions about either their own or their child’s executive functioning or self-regulation on their everyday behaviour. It is considered an ecologically valid alternative to lab-based assessments [54] and consists of 86 (parent) or 75 (self) questions related to everyday behaviours associated with executive function. It comprises eight subscales (Inhibit, Shift, Working memory, Plan/Organise, and Emotional Control, Initiate, Monitor, Organisation of Materials and Inhibitory Self-Control) that create an overall summary score (General Executive Composite). Higher scores indicate poorer EF. Visual and verbal memory will be assessed using ‘Dot locations’ and ‘Word pairs’ subtests of the Children’s Memory Scale ([CMS] < 16 years) and ‘Designs’ and ‘Verbal Paired Associates’ subtests of the Wechsler memory scale ([WMS] > 16 years). Attention will be measured using Conner’s Continuous Performance Test-III (CPT-III).

### Brain MRI

All participants will undergo MRI at baseline and, unless contraindicated, at study exit. MRI will be performed on a 3-T Siemens Prisma (Erlangen, Germany) at Great Ormond Street Hospital without sedation or contrast agents. MRI protocol of clinical sequences will consist of:

- T2‐weighted sequence
- Fluid‐attenuated inversion recovery (FLAIR) sequence
- Magnetic resonance angiography (MRA) sequence of the vessels of the Circle of Willis in the brain

These images will be read at baseline and exit by a neuroradiologist blinded to treatment arm who will diagnose SCI and any other clinically significant finding.

The MRI protocol for quantitative analysis blind to treatment arm includes:

- 3D T1‐weighted sequence for volumetric analysis and segmentation
- Diffusion tensor imaging (DTI) sequence for voxel‐wise white matter analysis and whole-brain tractography
- Arterial spin labelling (ASL) sequence for non‐invasive measurement of perfusion

MRI results will be assessed blind to treatment arm in two ways. Any change in radiological status (i.e. change from normal study, new or enlarged SCI, any other clinically significant change from baseline) will be recorded. For quantitative imaging analyses: (1) a longitudinal TBSS design will be implemented to show change in white matter metrics between treatment groups and correlations with change in haematological or cognitive outcomes; (2) global and regional cerebral blood flow maps will be calculated to show differences between treatment groups and correlations with change in haematological or cognitive outcomes; (3) volumetric segmentations of surface area, cortical thickness and grey matter volume will be calculated at each time point and differences will be compared between treatment groups as well as correlations with change in haematological or cognitive outcomes.

### Compliance

For the APAP arm, adherence to treatment will be formally assessed using specially designed software (Encore Pro™ data management software) and Smartcard which records and saves both qualitative and quantitative data on a single downloadable card. Compliance will be assessed regularly. Adequate adherence will be defined as use of the APAP for at least 4 h a night and at least 16 nights per month. A subgroup analysis of subjects using APAP for at least 4 h on the night before cognitive assessments will be evaluated

### Pain

Monitoring of daily pain will occur daily for two weeks at two separate time-points: baseline and end of study. Assessments will be performed using a Smartphone e-Diary (GoMed Solutions Inc), taking < 5 min per day to complete, which downloads anonymous data to a web-based database. This application collects information including whether or not pain was experienced and if it was, further questions on pain characteristics as follows: (1) current pain, using a 0–10 electronic numerical rating scale (e-NRS); (2) the highest e-NRS in past 24 h; (3) the lowest e-NRS in past 24 h; (4) the average-e-NRS in the past 24 h; (5) location of pain on a body outline diagram; (6) how much pain interfered with everyday activities in past 24 h; and (7) descriptors of pain to characterise its nature as neuropathic or nociceptive. The same information may also be collected on a paper form. In addition, pain characteristics and descriptors will be collected at the end of every month via a phone call using the Pain burden questionnaire [48]. Pain-related healthcare visits over the entire period, including admissions for pain (reported as expected serious adverse events), will be compared between the two arms to assess whether the intervention has an impact on hospital attendance and admission.

### Quality of life

Quality of life will be measured using the PedsQL, which contains a validated sickle module for use in adults and children [51]. Additionally, the utility measures CHU-9D and Euroqol EQ-5D [50] can be used for children and adults, respectively. These are preference-based quality of life measures that will be employed in estimating QALYs. As this is a study of an intervention for sleep, it is also important to use sleep questionnaires, specifically the Sleep Habits screener [46] and Epworth Sleepiness scale, [47] to document features such as snoring. This study provides the opportunity to assess the feasibility, acceptability and comparative performance of these measures in assessing SCA-related symptoms and quality of life.

### Statistical analysis

Analyses will be performed in accordance with the intention-to-treat principle, though a ‘per-protocol’ subgroup with adequate treatment adherence will be defined before database lock. The primary analysis of change in cancellation time will be based on an ANCOVA model containing the baseline cancellation score and minimisation factors as fixed covariates. A sensitivity analysis excluding the minimisation factors will also be performed. Pre-specified subgroups include the minimisation factors and the subgroup of individuals with adequate APAP compliance on the night before cognitive testing. Continuous variables that are collected at baseline and six months will be analysed using similar models. Pain and other outcomes with multiple collection points will be analysed using mixed models for repeated measures, based on observed data and including the fixed, categorical effects of treatment, minimisation factors, visit and treatment-by-visit interaction, as well as the continuous, fixed covariates of baseline and baseline-by-visit interaction. An unstructured covariance will be used to model the within-patient errors if possible; compound symmetry will be used otherwise. Binary outcomes will be analysed using logistic regression, including the minimisation variables as fixed covariates.

The overall significance level of 5% will be allocated for the primary analysis. Type 1 error adjustments for secondary outcomes will not be made and analyses beyond the primary outcome should be interpreted with caution.

### Health economic analysis

Detailed information on all resources required for both arms, including family born costs, productivity losses and/or out of school days for children, and costs from the health provider’s (NHS) perspective will be collected. Secondary care resource use will be collected from hospital records. Resources identified will be costed using appropriate local and national unit cost data to provide estimates of the cost incurred during the intervention.

The preference-based generic Health Related Quality of Life (HRQoL) measures CHU-9D for children and EQ-5D for adults will be used to enable estimation of QALYs for both arms. The sensitivity of these measures will be compared to other disease specific outcomes within the trial. Missing data will be examined as to whether cases with missing data are similar to those with full economic data in a similar manner to the main trial analysis.

Descriptive statistics will be presented for main cost outcomes and HRQoL measures in terms of QALYs; at each occasion inferences will be made and conclusions will be drawn, allowing identification of the best way of measuring and collecting data for the full cost-effectiveness analysis within further trials.

## Discussion

Despite the wide availability of APAP, the well-known impact of SCD on cognitive development and pain, and the growing body of evidence for higher rates of OSA in this patient population, little has been done to evaluate the impact of an OSA intervention for SCD. The results of this study will inform clinicians about the utility of APAP in improving three domains that are important to patients: cognitive function; pain; and quality of life. The primary endpoint, processing speed, can have a widespread impact on domains of cognitive functioning and everyday life. Since there may be practice effects on some cognitive tests, performance may improve in both arms of the trial [18], which necessitates the use of a control group. We foresee that there will be improvements in cognitive means for both arms, but that the treatment group will show statistically greater improvements compared to the control group.

The natural history of sleep-disordered breathing in young children in the general population is to improve and a large randomised controlled trial of adenotonsillectomy did not find a statistically significant improvement in an executive function endpoint [55]. For endpoints including cognition and quality of life, the relative contributions of nocturnal desaturation, which particularly affects patients with sickle cell disease, and sleep apnoea with arousals are hotly debated. For patients with sickle cell disease, much less is known about the natural history of sleep-disordered breathing; there is no clinical consensus about where the threshold might be for an effect on cognition, pain or quality of life. Based on our previous findings that many patients with sickle cell disease have minimum oxygen saturation overnight, lower than the minimum documented in healthy asymptomatic children in the general population, we have elected to recruit both patients with evidence for obstructive sleep apnoea and those with more severe desaturation into this trial. This decision will allow comparisons of the magnitude of benefit across these groups.

### Trial status

The first participant was randomised on 23 March 2016. As of 30 March 2017, 30 have been randomised in the paediatric cohort and 28 in the adult cohort.

## Abbreviations

ANCOVA: Analysis of covariance
APAP: Auto-adjusting continuous positive airways pressure
ASL: Arterial spin labelling
BRIEF: Behavior Rating Inventory of Executive Function
CHU-9D: Child Health Utility-9D
CMS: Children’s Memory Scale
CPAP: Continuous positive airways pressure
CPT-III: Conner’s Continuous Performance Test-III
D-KEFS: Delis-Kaplan tests of Executive Function
DTI: Diffusion tensor imaging
EF: Executive function
e-NRS: Electronic numerical rating scale
EQ-5D: Euroqol-5D
FLAIR: Fluid‐attenuated inversion recovery
HbSS: Haemoglobin SS
HPLC: High performance liquid chromatography
HRQoL: Health-related quality of life
IEF: Isoelectric focusing
MRA: Magnetic resonance angiography
MRI: Magnetic resonance imaging
MS: Mass spectrometry
NHS: National Health Service
NOT: Nocturnal Oxygen Therapy
OAHI: Obstructive apnoea hypopnoea index
OSA: Obstructive sleep apnoea
PEDS-QL: Pediatric Quality of Life Inventory
POMS: Prevention of morbidity in sickle cell disease
QALY: Quality-adjusted life year
RD: Radial diffusivity
SCA: Sickle cell anaemia
SCI: Silent cerebral infarction
SpO2: Peripheral oxygen saturation
TBSS: Tract-based spatial statistics
WAIS-IV: Wechsler Adult Intelligence Scale-IV
WASI: Wechsler Abbreviated Scale of Intelligence
WISC-IV: Wechsler Intelligence Scale for Children-IV
WMS: Wechsler memory scale
